# Melatonin attenuates angiotensin II-induced abdominal aortic aneurysm through the down-regulation of matrix metalloproteinases

**DOI:** 10.18632/oncotarget.15093

**Published:** 2017-02-04

**Authors:** Jing Kong, Ya Zhang, Shanshan Liu, Hongxuan Li, Shangming Liu, Jingjing Wang, Xiaoteng Qin, Xiuxin Jiang, Jianmin Yang, Cheng Zhang, Wencheng Zhang

**Affiliations:** ^1^ The Key Laboratory of Cardiovascular Remodeling and Function Research, Chinese Ministry of Education and Chinese Ministry of Health, The State and Shandong Province Joint Key Laboratory of Translational Cardiovascular Medicine, Qilu Hospital of Shandong University, Jinan, Shandong, China; ^2^ Key Laboratory of the Ministry of Education for Experimental Teratology, Department of Histology and Embryology, Shandong University School of Medicine, Jinan, China; ^3^ Institute of Pathology and Pathophysiology, Shandong University School of Medicine, Jinan, China; ^4^ Department of General Surgery, Virtual Laboratory, Qilu Hospital of Shandong University, Jinan, China

**Keywords:** abdominal aortic aneurysm, melatonin, HuR, MMP2, MMP9, Pathology Section

## Abstract

Abdominal aortic aneurysm (AAA) affects more than 5% of the population in developed countries and the pharmacotherapies for AAA are limited. Here, we explored whether melatonin regulates the development of AAA. In smooth muscle cells, melatonin treatment decreases angiotensin II-induced matrix metalloproteinase 2 (MMP2) and MMP9 expression. Human antigen R (HuR) could bind with the adenylateuridylate-rich elements of MMP2 and MMP9 mRNAs 3′ untranslated region, resulting in the increased stability of MMP2 and MMP9 mRNAs. HuR is required for angiotensin II-induced MMP2 and MMP9 expression. Moreover, melatonin suppresses angiotensin II-induced HuR expression through inhibiting NF-?B signaling, leading to decreased MMP2 and MMP9 levels. Finally, melatonin attenuates the development of AAA in ApoE^−/−^ mice infused with angiotensin II *in vivo*. These data support a role of HuR in the development of AAA and possible therapeutic roles for melatonin and/or HuR inhibition in AAA.

## INTRODUCTION

Abdominal aortic aneurysm (AAA), defined as abdominal aortic diameter ≥ 30 mm or increased normal aortic diameter by 50%, affects more than 5% of the population in developed countries [1]. After aortic dilation, rupture of AAA can occur with associated mortality of >80% [2]. Unfortunately, pharmacotherapies for AAA are limited currently. Thus, it is necessary to clarify the molecular mechanism of AAA and develop effective medical treatments that inhibit AAA progression in the world.

The pathogenesis of AAA is characterized by the degradation of the extracellular matrix (ECM) by the increased generation of reactive oxygen species (ROS), matrix metalloproteinases (MMPs) and inflammatory reactions [3]. MMPs digest both collagen and elastin, elevated levels of these MMPs have been shown to occur in aneurysmal aortic wall compared with normal aorta [4]. In animal models of AAA, genetic and pharmacological inhibition of MMPs suppressed aneurysm formation [5, 6]. Smooth muscle cells (SMCs) mainly express MMP2 and MMP9, which play a pivotal role during the progression of AAA [7].

Melatonin is a product of tryptophan metabolism and secreted by the pineal gland following a circadian rhythm and regulates diverse physiological processes [8]. Melatonin produces its biological effects through two main G-protein-coupled high-affinity melatonin receptors 1 and 2 (MT1 and MT2) [9]. Melatonin influences blood pressure, myocardial contractility and increases the antioxidant reserve [10]. More importantly, impaired melatonin production was reported in various cardiovascular pathologies including ischemic heart disease, hypertension with non-dipper pattern, or in patients after acute myocardial infarction [11–13], suggesting the protective roles of melatonin in the cardiovascular system. However, its role in AAA development has not been elucidated. In the present study, we examined the role of melatonin in AAA induced by angiotensin II (AngII) treatment in ApoE^−/−^ mice. The underlying mechanism was also explored. We believe that our findings may provide a new medical therapy for AAA.

## RESULTS

### Melatonin treatment inhibits AngII-induced MMP2 and MMP9 expression

**Figure 1:**
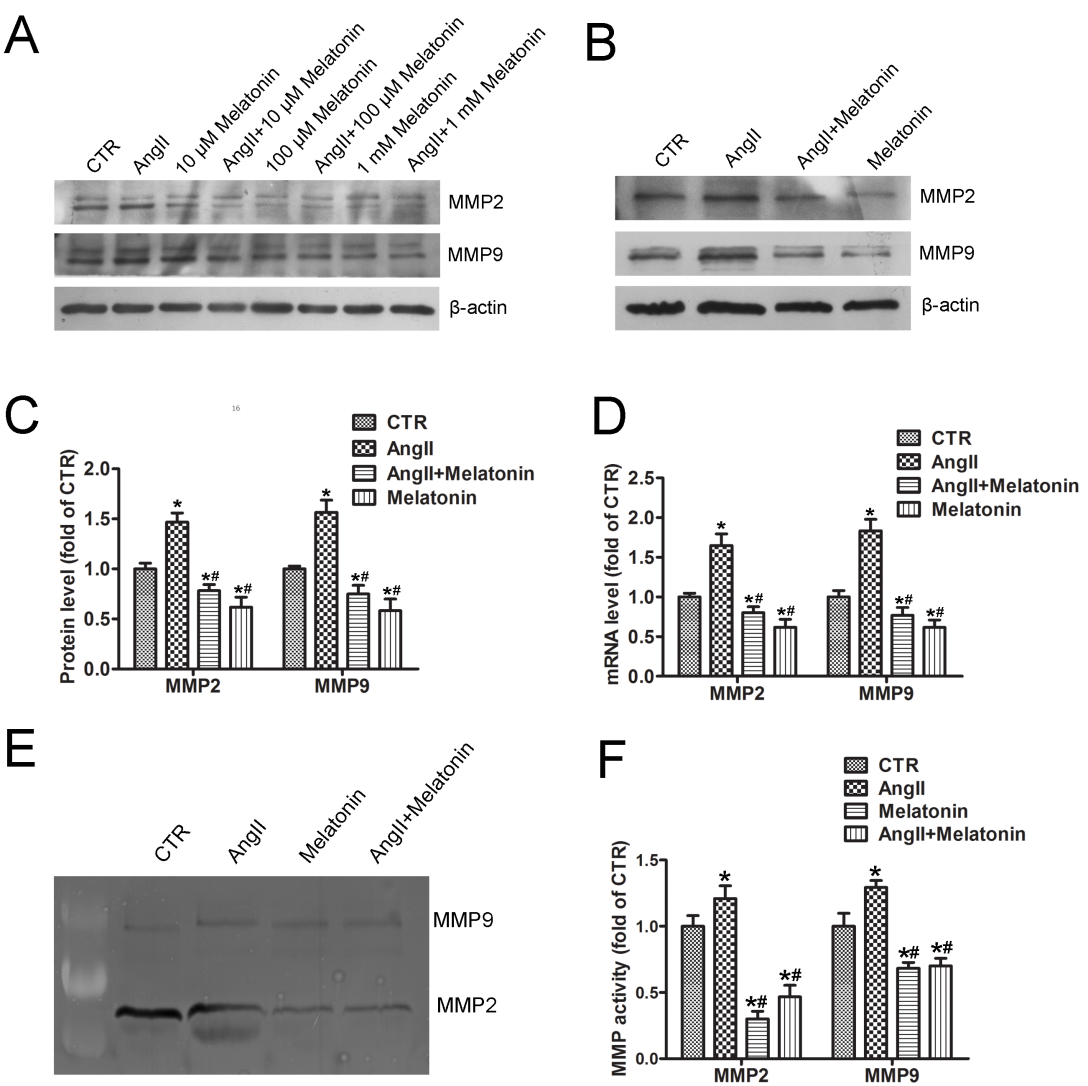
Melatonin treatment inhibits AngII-induced MMP2 and MMP9 expression **A**. MOVAS cells were pretreated with different concentration of melatonin (from 10 μM to 1 mM) for 4 hours followed by AngII treatment (1 μM) for 24 hours. Western blot was performed to detect MMP2 and MMP9 expression. **B**. MOVAS cells were pretreated with or without 100 μM melatonin followed by AngII treatment and western blot. **C**. and **D**. Quantitative analysis of MMP2 and MMP9 protein (C) and mRNA (D) levels in SMCs (*n* = 4). *p < 0.05 *vs* CTR group; #*p* < 0.05 *vs* AngII group. **E**. and **F**. Gelatin zymography of MMP2 and MMP9 activity (E) and their quantitative analysis (*n* = 4) (F). *p < 0.05 *vs* CTR group; #*p* < 0.05 *vs* AngII group.

Vascular SMC-derived MMP2 and MMP9 are considered as key factors in extracellular matrix degradation that is crucial for AAA development and aortic rupture [14]. To assess whether melatonin affects AngII-induced MMP2 and MMP9 expression, MOVAS cells were pretreated with different concentration of melatonin (from 10 μM to 1 mM) for 4 hours followed by AngII treatment overnight. Western blot analysis indicated that melatonin inhibited AngII-induced MMP2 and MMP9 expression in dose-dependent manner (Figure 1A). In the subsequent experiments, we selected 100 μM as the treatment concentration of melatonin. 100 μM melatonin pretreatment attenuated AngII-induced MMP2 and MMP9 protein expression (Figure 1B and 1C). More importantly, basal MMP2 and MMP9 protein levels were also inhibited by melatonin. Real-time PCR results showed that melatonin suppressed mRNA levels of MMP2 and MMP9 both in basal and AngII treatment (Figure 1D), suggesting that melatonin may decrease mRNA stability of MMP2 and MMP9 resulting in reduced protein levels. Moreover, AngII increased the gelatinase activity of MMP2 and MMP9, which was inhibited by melatonin pretreatment (Figure 1E and 1F).

### MMP2 and MMP9 are targets of Human antigen R (HuR)

**Figure 2:**
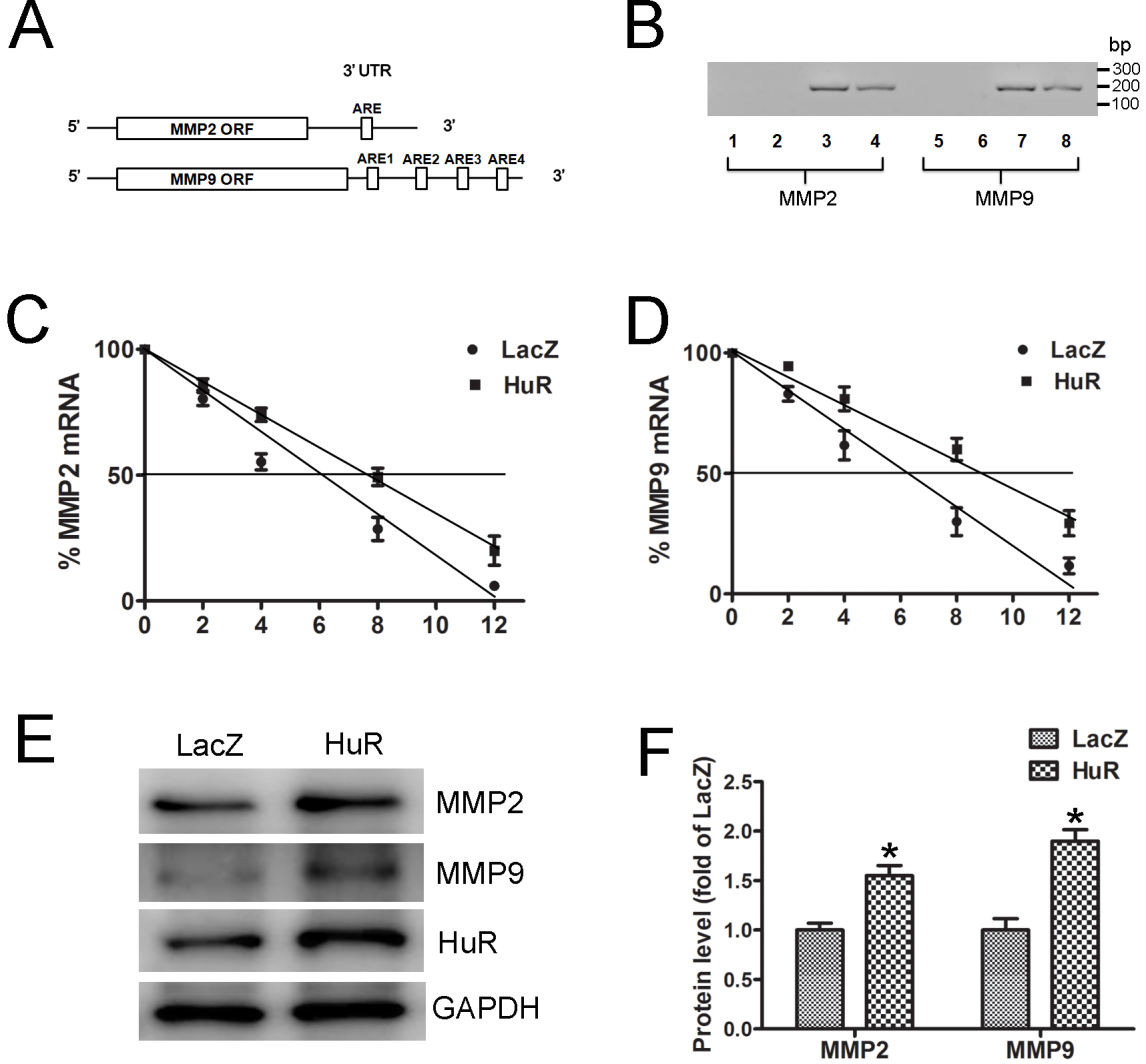
MMP2 and MMP9 are targets of HuR **A**. Schematic representations of predicted AREs in the 3’ UTR of MMP2 and MMP9 mRNAs are depicted. **B**. RNA immunoprecipitation was carried out with the anti-HuR antibody or control IgG. Lanes 1 and 5, no template PCR control; lanes 2 and 6, IgG RNA immunoprecipitation; lanes 3 and 7, Anti-HuR RNA immunoprecipitation; and lanes 4 and 8, 10% input. **C**. and **D**. MOVAS cells were transfected with LacZ or HuR plasmid for 24 hours and then treated with actinomycin D (5 μg/mL). The levels of MMP2 (C) and MMP9 (D) mRNAs were determined by real-time PCR (*n* = 4). **E**. Western blot analysis to detect MMP2 and MMP9 expression in MOVAS cells after LacZ or HuR transfection. **F**. Quantitative analysis of MMP2 and MMP9 protein levels in SMCs (*n* = 4). *p < 0.05 *vs* LacZ.

To better understand the regulation of MMP2 and MMP9 expression by melatonin, we examined their 5’ untranslated region (UTR) and 3’ UTR to assess whether MMP2 and MMP9 may be targets of posttranscriptional modification. We identified one and four conserved adenylate-uridylate-rich elements (AREs) in the 3’ UTR of MMP2 and MMP9 mRNA separately (Figure 2A). HuR is an RNA-binding protein that increases the stability of ARE-containing transcripts [15]. The ability of HuR to bind with the 3’ UTR of MMP2 and MMP9 mRNAs was assessed. RNA immunoprecipitation with either an anti-HuR antibody or control IgG showed that HuR binds to MMP2 and MMP9 mRNAs (Figure 2B).

Next, we examined the effects of HuR on the stability of endogenous MMP2 and MMP9 mRNA. MOVAS cells were transfected with HuR or LacZ plasmids and then treated with actinomycin D, a transcriptional inhibitor. The half-life of MMP2 mRNA was increased from 6 to 8 hours after HuR transfections (Figure 2C), similarly HuR overexpression increased the half-life of MMP9 mRNA from 6.5 to 9 hours (Figure 2D), indicating that HuR can enhance MMP2 and MMP9 mRNAs stability. Moreover, HuR overexpression increased MMP2 and MMP9 protein levels (Figure 2E and 2F). Therefore, MMP2 and MMP9 are targets of HuR.

### HuR is required for AngII-induced MMP2 and MMP9 expression

**Figure 3:**
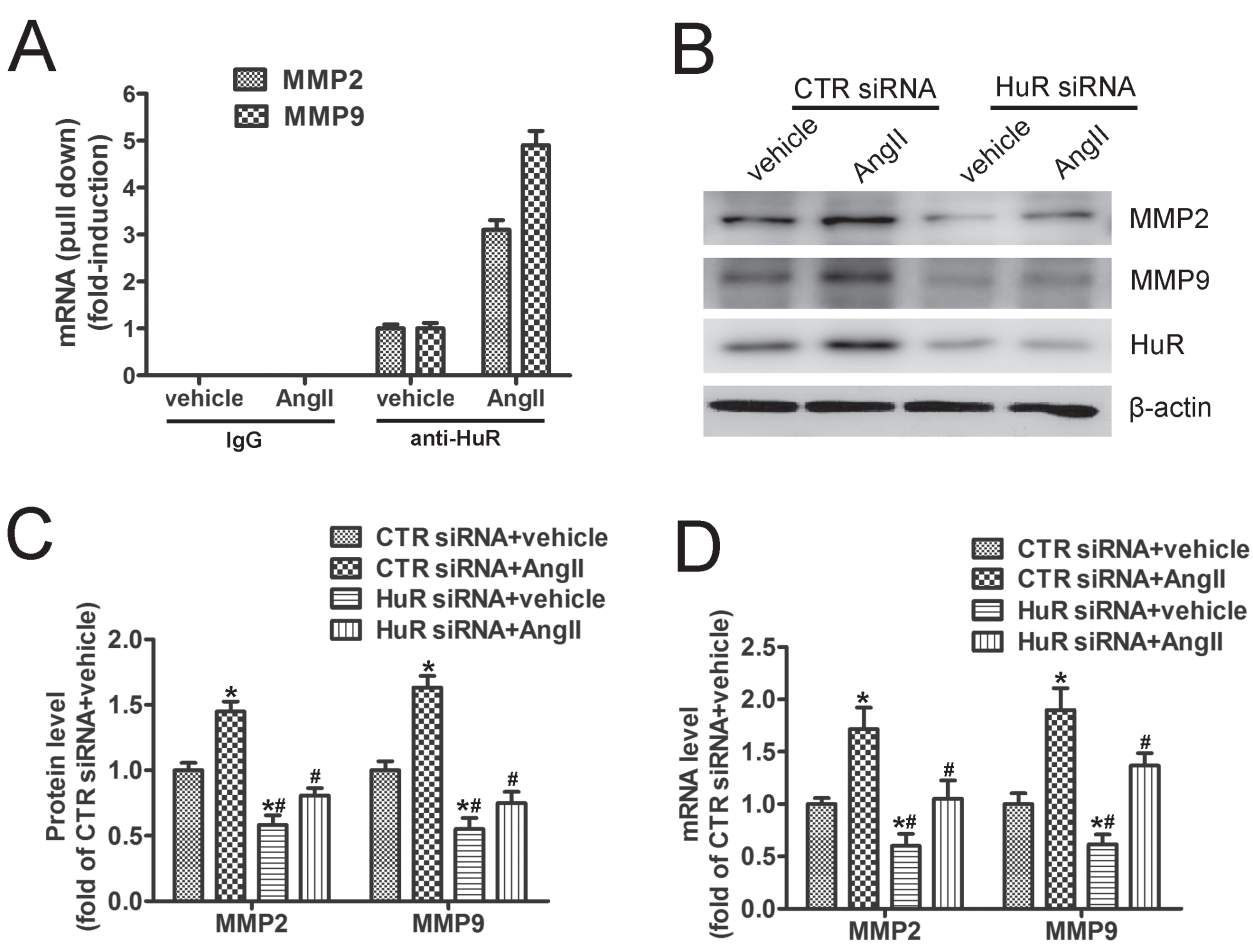
HuR is required for AngII-induced MMP2 and MMP9 expression **A**. Pull-down assay from MOVAS cells homogenates treated for 24 hours with either vehicle or with AngII (1 μM), which were immunoprecipitated with anti-HuR or with IgG. The RNA-bound by HuR was harvested and subjected to real-time PCR by using either MMP2- or MMP9-specific primers. Amounts of input RNA added to the immunoprecipitation reaction mixture was normalized by assessment of GAPDH levels from input RNA by real-time PCR (not shown). Data are depicted as -fold induction compared with vehicle treated cells. **B**. MOVAS cells were transfected with control or HuR-specific siRNA for 48 hours, followed by AngII treatment and western blot analysis. **C**. and **D**. Quantitative analysis of MMP2 and MMP9 protein (C) and mRNAs (D) levels in SMCs (*n* = 4). *p < 0.05 *vs* CTR siRNA+vehicle group; #*p* < 0.05 *vs* CTR siRNA+AngII group.

As demonstrated above, MMP2 and MMP9 are targets of HuR. To assess the roles of HuR in AngII-induced MMP2 and MMP9 expression, pull-down assay from MOVAS cells treated with either vehicle or with AngII was performed. AngII treatment induced overall three-fold higher HuR-MMP2 or five-fold higher HuR-MMP9 mRNA content when compared with vehicle treatment (Figure 3A), indicating that AngII increases HuR-binding to MMP2 and MMP9 mRNAs. Moreover, HuR knockdown with siRNA suppressed AngII-induced MMP2 and MMP9 expression (Figure 3B and 3C), suggesting that HuR is required for AngII-induced MMP2 and MMP9 expression. Besides, HuR knockdown also reduced mRNA levels of MMP2 and MMP9 both in basal and AngII treatment (Figure 3D). It is notable that AngII also increased MMP2 and MMP9 expressions in the HuR knockdown cells (Figure 3B)*,* suggesting that AngII can increase the levels of MMP2 and MMP9 in HuR-dependent and HuR-independent manners. Taken together, these data indicate that AngII induce HuR to bind to and stabilize the endogenous MMP2 and MMP9 mRNA transcripts, thus resulting in elevated MMP2 and MMP9 protein levels.

### Melatonin inhibits AngII-induced HuR expression

**Figure 4:**
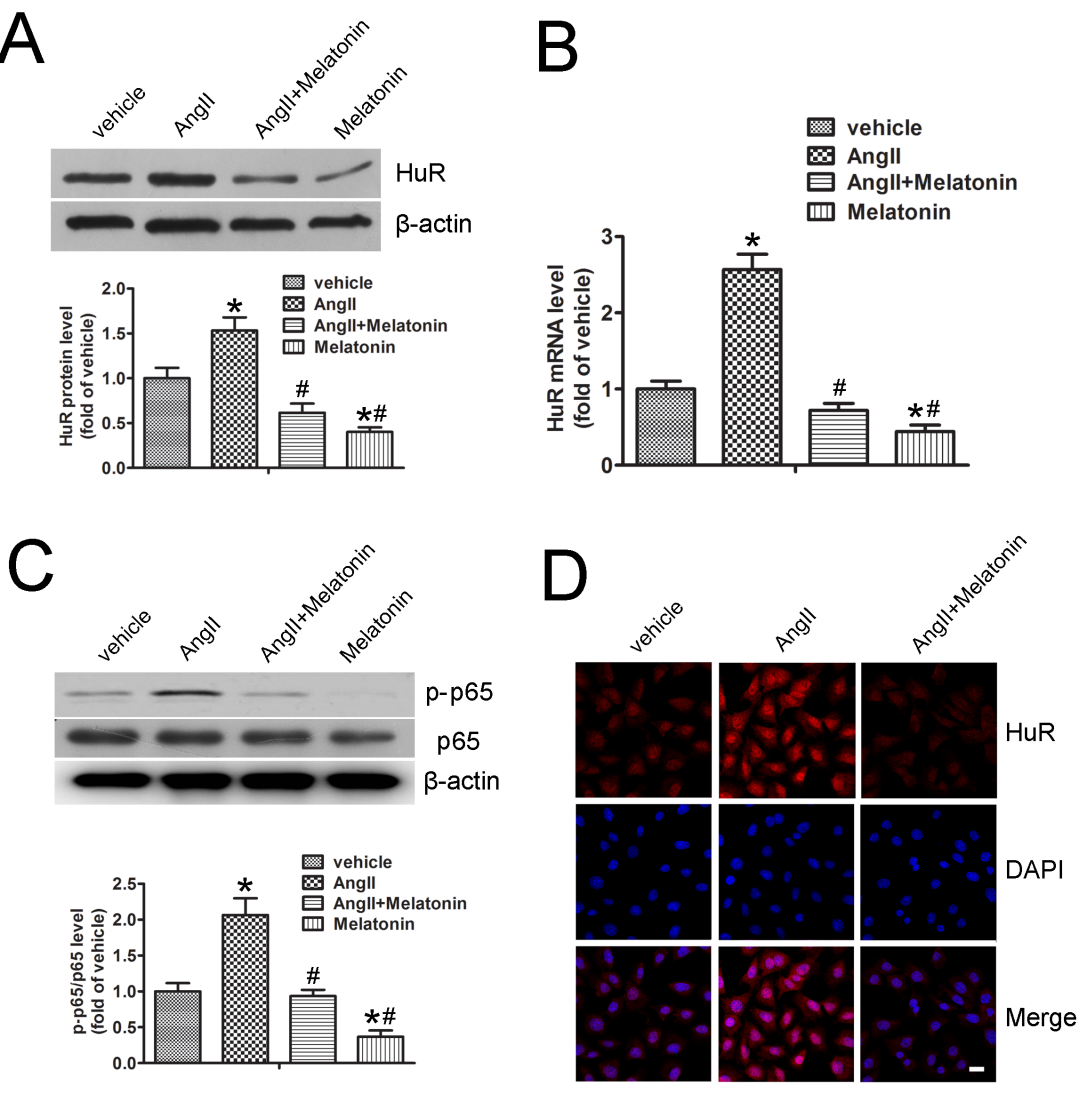
Melatonin inhibits AngII-induced HuR expression **A**. MOVAS cells were pretreated with or without melatonin followed by AngII treatment. Protein expression of HuR and quantitative analysis were performed (*n* = 4). **B**. Quantitative analysis of HuR mRNA levels in SMCs (*n* = 4). **C**. MOVAS cells were pretreated with or without melatonin followed by AngII treatment. Protein expression of phospho-p65/total p65 and quantitative analysis were performed (*n* = 4). **D**. Immunostaining was performed to determine the localization of HuR (red) in MOVAS cells treated with melatonin and AngII. Nuclei were stained with DAPI (blue). Scale bar, 10 μm. *p < 0.05 *vs* vehicle group; #*p* < 0.05 *vs* AngII group.

Previous study reported that AngII promoted an increase in nucleo-cytoplasmic HuR shuttling in human mesangial cells [16]. However, in SMCs, AngII increased HuR protein levels (Figure 4A), while melatonin pretreatment inhibited basal or AngII-induced HuR expression (Figure 4A). Moreover, AngII induced a 2.56-fold increase in HuR mRNA levels, which was reduced by melatonin pretreatment (Figure 4B). As p65/RelA can bind to a putative NF-κB binding site in the HuR promoter [17], we detected whether melatonin can affect NF-κB signaling in SMCs. AngII increased phospho-p65 Ser536, which is a marker for NF-κB activation. Melatonin pretreatment inhibited AngII-induced p65 phosphorylation (Figure 4C). In cells, only cytoplasmic HuR can bind to the 3’ UTR of target mRNAs and enhance their stability. Immunofluorescence analysis revealed that AngII increased cytoplasmic HuR levels, which were reduced by melatonin (Figure 4D). Taken together, these data suggest that AngII increases HuR expression through NF-κB signaling and subsequent HuR binding to MMP2 and MMP9 mRNAs, resulting in elevated MMP2 and MMP9 mRNAs stability and protein levels. Melatonin inhibits NF-κB signaling and AngII-induced HuR expression which lead to decreased MMP2 and MMP9 levels.

### Melatonin attenuates AngII-induced AAA *in vivo*

**Figure 5:**
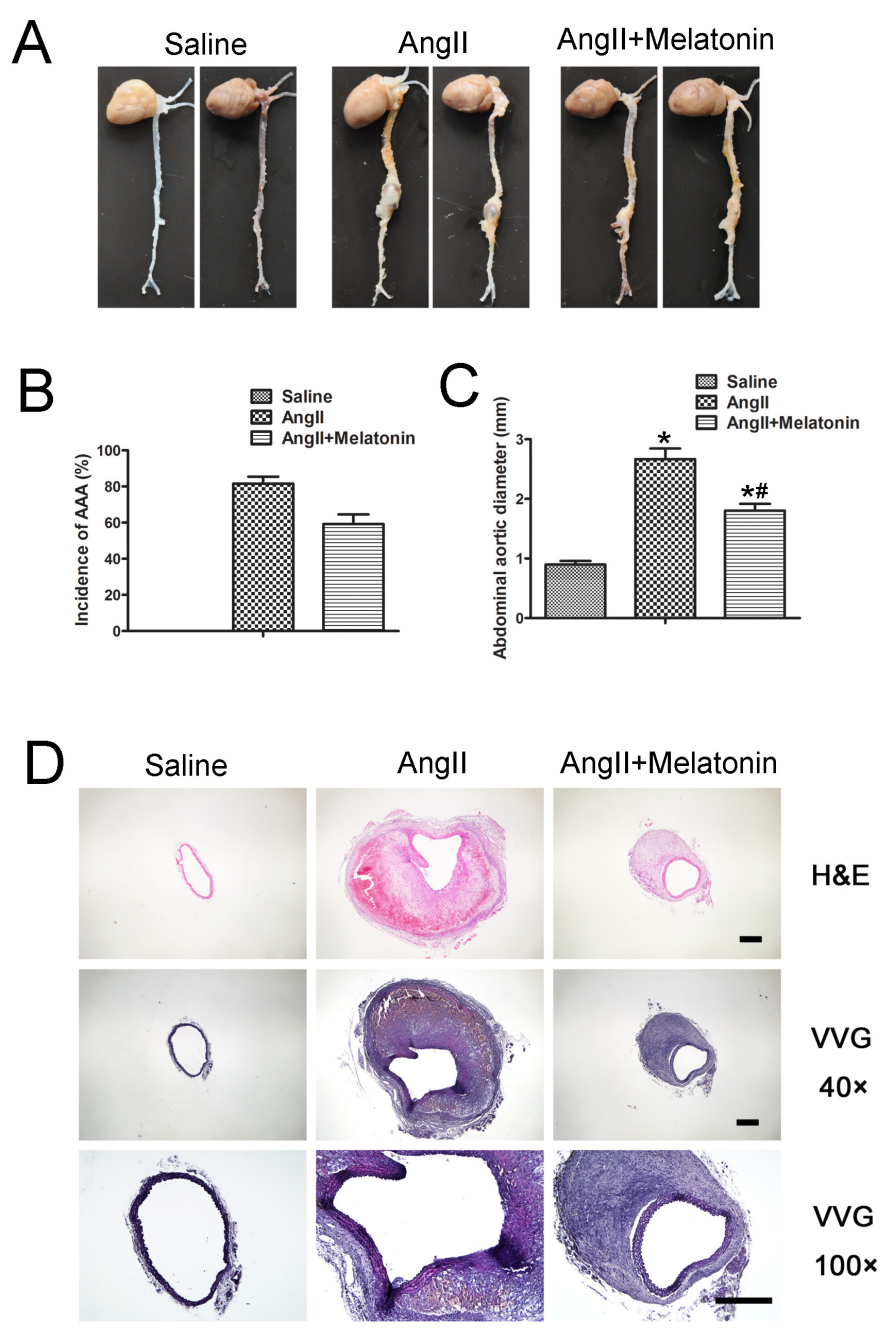
Melatonin attenuates AngII-induced AAA *in vivo* **A**. Representative photographs of abdominal aortic specimens in three groups of mice who received treatment with saline, AngII and AngII plus melatonin, respectively. **B**. Incidence of AAA in three groups of mice. **C**. Maximal abdominal aortic diameters in three groups of mice. *n* = 30, *p < 0.05 *vs* Saline group; #*p* < 0.05 *vs* AngII group. **D**. Representative H&E and VVG staining in three groups of mice. Scale bar, 500 μm.

To assess whether melatonin affects AngII-induced AAA *in vivo*, ApoE^−/−^ mice were infused subcutaneously with AngII by osmotic minipumps and with or without melatonin. After 28 days, the aortic segments from the aortic arch to the renal arteries were examined. The incidence of aortic aneurysm was 0% in the saline group, 82% in the AngII group, 60% in the AngII plus melatonin group (Figure 5A and 5B). As well, the maximal diameter of the abdominal aorta was significantly reduced in the AngII plus melatonin group compared with AngII group (Figure 5C).

Hematoxylin and eosin and Verhoff-Van Gieson staining revealed that AngII infusion caused positive remodeling of the abdominal aorta, breakdown of the aortic media and adventitia and destruction and discontinuity of elastin fibers in ApoE^−/−^ mice (Figure 5D). Compared with the AngII group, these pathological changes induced by Ang II were largely attenuated in the Ang II plus melatonin group (Figure 5D). Taken together, melatonin treatment attenuates AngII-induced AAA formation in ApoE^−/−^ mice *in vivo*.

### Melatonin suppresses HuR, MMP2 and MMP9 expression *in vivo*

**Figure 6:**
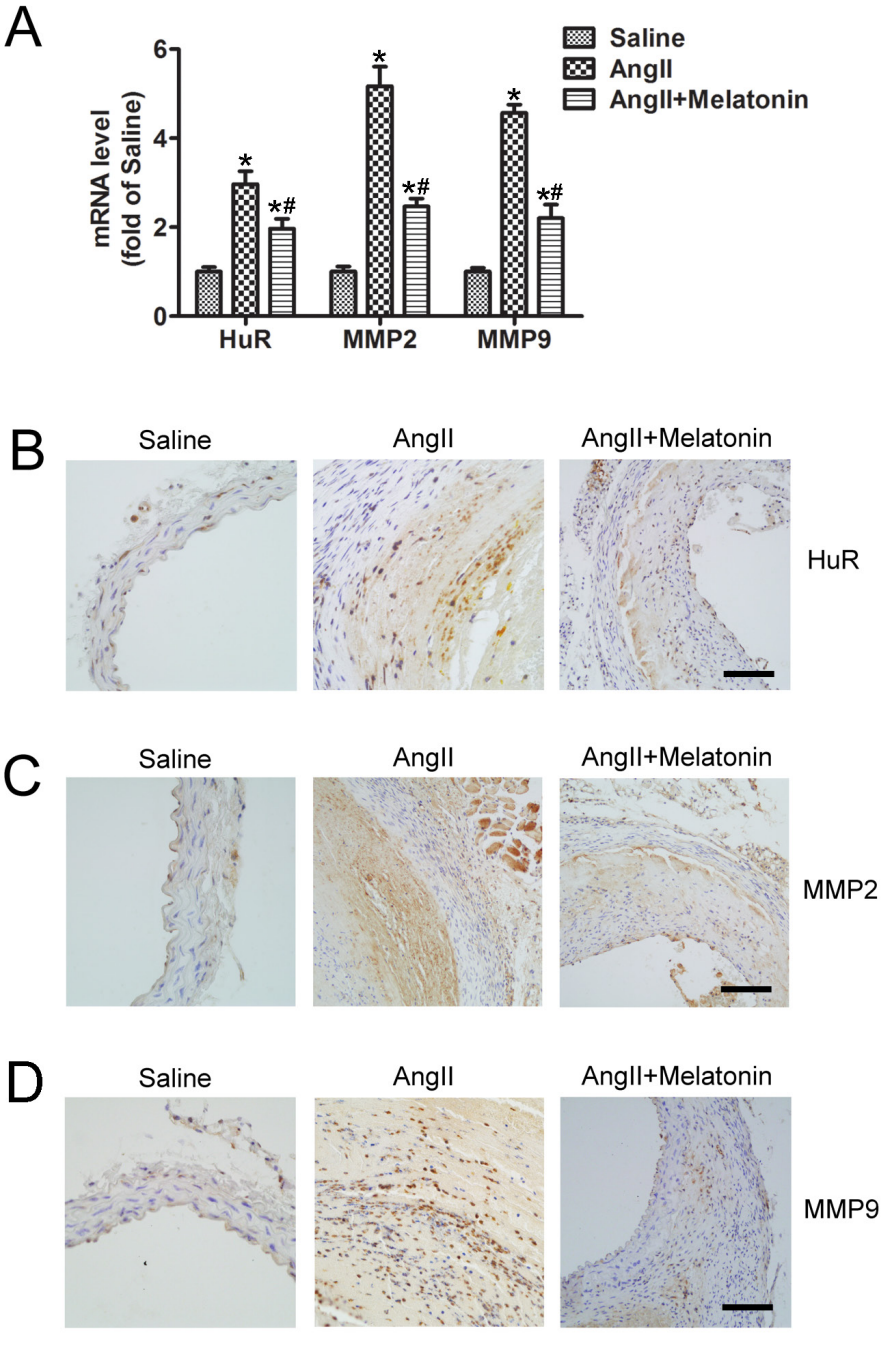
Melatonin suppresses HuR, MMP2 and MMP9 expression *in vivo* **A**. Quantitative analysis of HuR, MMP2 and MMP9 mRNAs levels in abdominal aortic tissues from three groups of mice who received treatment with saline, AngII and AngII plus melatonin (*n* = 5). *p < 0.05 *vs* Saline group; #*p* < 0.05 *vs* AngII group. B to D, Representative immunostaining of HuR (**B**.) MMP2 (**C**.) and MMP9 (**D**.) in mice who received treatment with saline, AngII and AngII plus melatonin. Scale bar, 100 μm.

The mRNAs expression in the aortic tissues assessed by real-time PCR indicated that mRNA levels of HuR, MMP2 and MMP9 were significantly increased in AngII group compared with saline group, and melatonin treatment reduced their mRNA levels (Figure 6A). Meanwhile, the protein levels of HuR, MMP2 and MMP9 were assessed by immunohistochemistry. AngII infusion increased HuR, MMP2 and MMP9 protein levels, which were inhibited by melatonin treatment (Figure 6B-6D). Therefore, melatonin suppressed HuR, MMP2 and MMP9 expression *in vivo,* which were consistent with data from cells.

## DISCUSSION

In this study, we demonstrated that melatonin protects against AngII-induced AAA in ApoE^−/−^ mice. Melatonin treatment significantly reduces the incidence of AAA and aortic dilatation in AAA model *in vivo*. Mechanically, it attenuates AngII-induced HuR expression which results in reduced MMP2 and MMP9 mRNAs stability and protein levels. Our findings suggest a new agent for AAA pharmacotherapy.

AAA may be caused by multiple reasons such as genetic factors, SMC apoptosis, ROS increase, inflammation and ECM degradation due to elevated MMPs activity [3]. Some signaling pathways such as NF-κB signaling have been implicated as causative in the development of AAA [18]. However, there is no effective pharmacological therapy to prevent or suppress AAA development and progression. In our study, intraperitoneal injection of melatonin markedly reduced the severity of AAA in AngII-infused ApoE^−/−^ mice through inhibiting HuR expression and subsequent decreased MMP2/9 mRNAs stability. In a recent report, melatonin inhibited AAA formation in elastase-perfused rat through its antioxidant property including reduced the levels of lipid peroxide, activities of NADPH oxidases and content of ROS [19]. Melatonin is often available as dietary supplement without need of medical prescription [8], what increases the chance of regular melatonin intake by patients suffering from AAA. Therefore, our findings are important and significant for the patients with AAA.

There was a significant increase in the levels of MMPs in AAA animal models and AAA patients’ specimens [7, 20]. Thus, it is important to explore the molecular mechanism underlying MMPs upregulation in the pathogenesis of AAA. It has been known that many transcription factors such as AP-2α, Smads and NF-κB are involved in the regulation of MMPs expression [21–23]. However, there is little knowledge about MMPs mRNA stability in the development of AAA. In this study, we demonstrated that HuR could bind to the 3’ UTR of MMP2 and MMP9 mRNAs, thus resulting in enhanced mRNAs stability. Our findings provide a new regulatory mechanism for MMPs expression. Besides, melatonin inhibited NF-κB signaling, which was reported to increase MMPs transcription [23]. Thus, melatonin was supposed to suppress MMPs expression at both the levels of mRNA transcription and mRNA stability. Moreover, AngII-induced cytoplasmic HuR accumulation was reported in human mesangial cells [16]. In the present study, AngII increases HuR expression through NF-κB signaling pathway in SMCs, which was consistent with previous report that NF-κB was a transcription factor of HuR [17]. In consideration of the vital roles of MMPs in AAA, our findings are highly significant in exploring the development of AAA.

Melatonin is an endogeneous indole compound, which is synthesized and secreted by the pineal body in vertebrates. Melatonin is widely used to counter transatlantic travel jet lag and insomnia. However, melatonin is less well known for its multiple and widespread physiological and pharmacological effects [24]. Previous studies have shown that melatonin is a powerful antioxidant and anti-inflammatory molecule [25, 26], with proven antihypertensive and lipid lowering effects [27, 28]. Moreover, melatonin can attenuate the development of diabetic complications and tumor progression [29, 30]. In this study, we found that melatonin treatment protected against AngII-induced AAA formation in ApoE^−/−^ mice. Thus, our findings may provide a new medical therapy for AAA, which amplify our understanding about the protective roles of melatonin in the cardiovascular diseases.

In conclusion, our study demonstrates that melatonin protects against AngII-induced AAA formation in ApoE^−/−^ mice. Mechanically, melatonin inhibits AngII-induced HuR expression though NF-κB signaling pathway, thus resulting in reduced MMP2 and MMP9 mRNAs stability and protein levels. Therefore, melatonin and/ or targeting HuR may serve as a potential therapeutic strategy for AAA.

## MATERIALS AND METHODS

### Cell culture and treatment

The mouse SMC line MOVAS cells were maintained in SMC medium (ScienCell, Carlsbad, CA, USA) containing 2% fetal bovine serum, 1% SMC growth supplement, 100 U/ml penicillin and 10 mg/ml streptomycin. Cells were seeded in multi-well plates at a density of 1.0×10^4^ cells/cm^2^. At confluence, cells were pretreated with melatonin at described dose for 4 hours followed by 1 μM AngII stimulation for 24 hours.

### Western blot

Protein extracts were prepared by lysing cells in Radio-Immunoprecipitation Assay (RIPA) lysis buffer from Santa Cruz Biotechnology (Santa Cruz, CA, USA). Cleared lysates were separated on 10% Tris-glycine gels followed by transfer onto nitrocellulose membranes. After blocking in 5% skim milk, blots were probed using primary antibodies including β-actin antibody (1:1000, Cell Signaling Technology, Danvers, MA, USA), HuR antibody (1:500, Millipore, Temecula, CA, USA), MMP2 antibody (1:500, Abcam, Cambridge, MA, USA), MMP9 antibody (1:500, Abcam, Cambridge, MA, USA), nuclear factor κB antibody (NF-κB p65, 1:1000, Cell Signaling Technology, Danvers, MA, USA) and phospho-p65 Ser536 (1:1000, Cell Signaling Technology, Danvers, MA, USA). After a wash and incubation with horseradish peroxidase (HRP)-conjugated secondary antibodies, protein bands were visualized by use of the chemiluminescent HRP substrate (Millipore, Temecula, CA, USA). The intensities of individual bands were measured by densitometry (Model GS-700, Imaging Densitometer; Bio-Rad, Hercules, CA, USA). All experiments were repeated for four times and the mean values derived.

### Quantitative real-time PCR

Total RNA was extracted from cells or tissues (kept at -80°C) by using Tri Reagent (Ambion, Austin, TX, USA), and 1 μg of RNA was reverse-transcribed into cDNA by using iScriptcDNA Synthesis Kits (Bio-Rad, Hercules, CA, USA). PCR amplification involved the SYBR PCR mix (Bio-Rad, Hercules, CA, USA). The oligonucleotide primer sequences used were for MMP2, 5’-ACCAAGGATATAGCCTATTCCT-3’ and 5’-GGCCAGTACCAGTGTCAGTA-3’; MMP9, 5’-GCTTAGATCATTCCAGCGTGCC-3’ and 5’-AGGTATAGTGGGACACATAGTGG-3’; HuR, 5’-ATGAAGACCACATGGCCGAAGACT-3’ and 5’-AGTTCACAAAGCCATAGCCCAAGC-3’; GAPDH, 5’-TTGTCAAGCTCATTTCCTGGTATG-3’ and 5’-GCCATGTAGGCCATGAGGTC-3’.

### RNA immunoprecipitation assays

The Magna RIP kit (Millipore, Temecula, CA, USA) was used for RNA immunoprecipitation assays. Briefly, whole-cell lysates were incubated at 4°C overnight with magnetic protein A/G beads previously precoated with 5 μg of either rabbit IgG or HuR antibody. Beads were washed and incubated with proteinase K buffer (30 minutes at 55°C), followed by RNA isolation from the immunoprecipitates and subsequently cDNA synthesis. Polymerase chain reaction was performed by use of MMP2 and MMP9 primers described in Quantitative Real-time PCR.

### Immunofluorescence

MOVAS cells were treated with AngII and with or without melatonin, then cells were fixed with cold actone, permeabilized with 0.1% Triton X100 and immune-stained with anti-HuR followed by incubation with Alexa Fluor 594 goat anti-rabbit IgG. Nuclei were stained with DAPI. Photos were taken under fluorescent microscope.

### Animals

Male ApoE^−/−^ mice (8-12 weeks old) were from Vital River Laboratories (Distributor of Jackson Laboratory, Beijing, China) and housed under specific pathogen-free conditions on a 12-hr light/12-hr dark cycle with food and water freely available. All ApoE^−/−^ mice were fed with a high fat diet (0.25% cholesterol and 15% cocoa butter) and divided randomly into three groups (*n* = 30 in each group). The control group received continuous subcutaneous infusion of saline for 28 days via an osmotic pump (Alzet, model 2004, Durect Corp., Cupertino, CA, USA), the AngII group received infusion of AngII (1.44 mg/kg/day, Lintai Biological Technology Company, Xi’an, China) plus intraperitoneal injection with 4% ethanol and the melatonin group received infusion of AngII (1.44 mg/kg/day) plus intraperitoneal injection of melatonin in 4% ethanol (30 mg kg^−1^ day^−1^, Sigma, St Louis, MO, USA) [31]. After 28 days of infusion, all ApoE^−/−^ mice were euthanized and their aortic tissues removed for further analysis. The animal experimental protocol was reviewed and approved by the Institutional Animal Care and Use Committee (IACUC) of Shandong University.

### Histology and immunohistochemical staining

Euthanized mice were perfused with saline to eliminate blood in the lumen, and the aortas of ApoE−/− mice were removed and fixed in 4% paraformaldehyde. The adventitia was removed the next day, and the maximal diameter of the abdominal aorta was measured.

The aortic segments from the aortic arch to the renal arteries were removed and embedded in OCT compound and frozen. Serial 5-μm thick sections were cut and collected on glass slides for histology. Corresponding sections on separate slides were visualized by hematoxylin and eosin (H&E) and Verhoeff-Van Gieson (VVG) staining for morphologic assessment. Immunohistochemistry involved use of the antibodies to assess the expression of HuR, MMP2 and MMP9 in the aortic tissues.

### Statistical analysis

All calculations involved use of SPSS v23 (SPSS Inc., Chicago, IL, USA). Data are represented as mean ± SD. Comparison of two groups of continuous data involved Student *t* test and multiple groups one-way ANOVA, followed by the Scheffe post-hoc test. Pearson correlation analysis was used for correlation analysis after D’Agostino-Pearson omnibus normality testing; otherwise, Spearman correlation analysis was used. Significance was determined at *p* < 0.05.

## Abbreviations

AAA: Abdominal aortic aneurysm
AngII: angiotensin II
MMP2: matrix metalloproteinase 2
MMP9: matrix metalloproteinase 9
HuR: Human antigen R
ECM: extracellular matrix
ROS: reactive oxygen species
SMC: smooth muscle cell
UTR: untranslated region
ARE: conserved adenylate-uridylate-rich element
